# Molecular Characterization of Aquaglyceroporine: A Novel Mutation in *LmAQP1* from *Leishmania major* (MRHO/IR/75/ER)

**Published:** 2019

**Authors:** Gilda ESLAMI, Maryam GHAVAMI, Ali Reza MORADI, Hamid NADRI, Salman AHMADIAN

**Affiliations:** 1. Research Center for Food Hygiene and Safety, Shahid Sadoughi University of Medical Sciences, Yazd, Iran; 2. Department of Parasitology and Mycology, Faculty of Medicine, Shahid Sadoughi University of Medical Sciences, Yazd, Iran; 3. Department of Medicinal Chemistry, Faculty of Pharmacy and Pharmaceutical Sciences Research Center, Shahid Sadoughi University of Medical Sciences, Yazd, Iran

**Keywords:** Aquaporin 1, *Leishmania*, Molecular dynamics simulation, Antimony

## Abstract

**Background:**

The first line treatment for cutaneous leishmaniasis is pentavalent antimony such as sodium stibogluconate (pentostam) and meglumine antimonite (glucantime). One of the most important ways to uptake the drug is by a trans-membrane protein, called aquaglyceroporin encoded by *Aquaglyceroprotein1* (*LmAQP1*). In this study, molecular characterization of *LmAQP1* was reported.

**Methods:**

*Leishmania major* (MRHO/IR/75/ER) promastigotes were cultured, and then DNA extraction and RNA extraction were done and followed by cDNA synthesis. Amplicons resulted from PCR and RT-PCR using specific primers were purified and sequenced. Molecular characterization was done by bioinformatically software such as BLST, ClustalW2, and RMSD.

**Results:**

Amplicons resulted from PCR and RT-PCR showed equal size in length. BLASTn analysis showed a point nucleotide change in *LmAQP1* gene that encoded 282-amino-acid long protein with a mutation at position 154 including replacement of alanine by threonine. The observed mutation in the interested gene was assessed using the above-mentioned software. The mentioned gene was submitted at GenBank, NCBI with accession number of KU514052.

**Conclusion:**

The functional prediction of the protein encoded from *LmAQP1* showed that the mentioned mutation could not affect the three-dimension structure, but it may modify the drug uptake potential of this important channel. Based on from *LmAQP1* role, it seems to be an appropriate candidate for drug development. According to search through internet, this is the first report of *LmAQP1* from *L. major* (MRHO/IR/75/ER).

## Introduction

Cutaneous leishmaniasis (CL) is the most common clinical form of leishmaniasis, usually caused by *Leishmania major* and *L. tropica* and *L. aethiopica* in the Old World (1). The first line treatment for CL is pentavalent antimony [Sb(V)] such as sodium stibogluconate (pentostam) and meglumine antimoniate (glucantime) (2). The important mechanism in *Leishmania* spp. responsible for Sb(V) transport is aquaglyceroporin (AQP) which is a membrane channel with six bilayer and two NPA (asparagines-proline–alanine) motifs (3–5). LmAQP1 is a mercurial-independent water channel and therefore is not inhibited by mercurite chloride. LmAQP1 channel transfers some other materials such as glycerol, glyceraldehyde, dihydroxyacetone, and sugar alcohols (6). Therefore, transporting of water and solute by LmAQP1 helps the parasite to face the osmotic challenges inside the sand fly invertebrate hosts (6). Therefore, LmAQP1 plays a specific role in *Leishmania* physiology and responsiveness to antimonial drugs.

Drug resistance or no response to antimonials in CL caused by *L. major* is one of the public health problems in endemic areas. The mechanisms of this failure are not yet clear, however, drug resistance might result from failure to drug uptake, efflux, and sequestration of active molecules (7–9). Down-regulation of *LmAQP1* is correlated with lower SbIII up-take and therefore low drug concentration within the cell (5). On the other hand, gene amplification of DNA segments has been observed in several *Leishmania* species selected for drug resistance (10, 11). Recently more studies in Iran have reported drug resistances in cutaneous leishmaniasis (12–14). Moreover, they mentioned to AQP1 as one of the main targets for antimonial resistance (12–14).

Understanding the genetic diversity of different *Leishmania* spp. is necessary to overcome ultimately current limitations in anti-parasitic drugs. Therefore, due to its involvement in a vast array of biological phenomenon, *LmAQP1* aimed at a target for investigation. To our knowledge, there is no report on *LmAQP1* gene sequence of *L. major* (MRHO/IR/75/ER), the *L. major* used for mass leishmanization and preparation of Old World experimental *Leishmania* vaccine and leishmanin (15).

In this study, the full-length sequence of the *LmAQP1* gene from *L. major* (MRHO/IR/75/ER) was reported, the gene sequence was also compared with *LmAQP1* from *L. major* Friedlin (AY567835).

## Materials and Methods

### Parasites

*L. major* (MRHO/IR/75/ER) was obtained from September 2015 to December 2016 from different research centers including, Center for Research and Training in Skin Diseases and Leprosy, Tehran, Iran and Research Center for Food Hygiene and Safety, Yazd, Iran. The promastigotes were cultured in Novy-McNeal-Nicolle medium, and subpassaged in RPMI 1640 medium (Sigma, USA) supplemented with 10% Fetal Calf Serum (FCS, Sigma), 100 U/ml penicillin G, and 100 μg/ml streptomycin at 26±1 °C. This step was done more than triplicate.

### DNA extraction

DNA from promastigotes (10^6^/ml) was extracted according to the method (16) with a minor modification. Briefly, lysis was done using a NET buffer (NaCl 25mM, EDTA pH 8 10mM, Tris-base pH 7.6 20 mM) supplied by SDS with end concentration of 1%. Purification was performed using phenol-chloroform-isoamyl alcohol. Precipitation was done by cold absolute ethanol and ammonium acetate 3M pH 5. Then, washing was done with cold ethanol 75%. The DNA sample was quantified and analyzed by spectrophotometer and agarose gel electrophoresis.

### RNA extraction

Promastigotes (10^6^/ml) were used for RNA extraction using RNX™ solution (CinnaGen) according to the manufacturer's instruction under RNAse free condition. The extracted sample was quantified and analyzed by spectrophotometer and agarose gel electrophoresis.

### cDNA synthesis

In order to synthesis cDNA, RevertAid^™^ First Strand cDNA Synthesis Kit (#K1621, Fermentas) was used according to the manufacturer instruction.

### Primers

The specific primer pair was designed based on the nucleotide sequence data of *LmAQP1* gene (AY567835) obtained from GenBank. The sequences of designed sense and antisense primers used in this study were 5′-GCGAAGTACACCCCTTTT -3′ and 5′-GTTTGTACGCCCAGGAAA -3′ with the product length of 1020bp. The primer pair was designed in a way that the upstream and downstream of the mentioned gene was amplified.

### PCR and RT-PCR

Amplification was performed by either *L. major* genomic DNA or cDNA as the template. The master mix was contained 10 mM Tris-HCl pH 8.3, 50mM KCl, 1.5mM MgCl2, 0.2mM each dNTPs, 5 pmol each primer and 1 U *Pfu* DNA polymerase (Fermentas). Thermal cycling was applied as 94° C for 5 min as initial denaturation, followed by 30 cycles with 94 °C for 45 sec, 52 °C for 45 sec and 72 °C for 45 sec. The final one cycle of 72 °C applied for 5 min as final extension. The amplicon was analyzed using agarose gel electrophoresis. This step was repeated more than triplicate.

### Sequencing

The amplified fragments were purified using High Pure PCR Product Purification Kit (#11732668001, Roche) and sequenced. The sequencing was done triplicate with both forward and reverse primers.

### Characterization and molecular analysis

Molecular characterization of the *LmAQP1* gene and its encoded protein were carried out using Basic Local Alignment Search Tool (BLAST) analysis, secondary structure prediction and infrastructural analysis (17, 18). The obtained sequence of *LmAQP1* gene was aligned with the one in *L. major*, Friedlin (AY567835).

### 3D structure analysis

To predict three-dimensional (3D) structure, the mentioned sequence from *L. major* Friedlin strain as a template and the one from Iranian standard *L. major* (MRHO/IR/75/ER) strain sequences were taken to IT-ASSER database server. This server provided alignment, additional with 3D structure, and then, the prediction was continued using MOE software for more explanation of the active site and the pore of channel. Moreover, RMSD was calculated and the distance between each atom was measured.

## Results

### Gene sequence analysis of LmAQP1

**Fig. 1:**
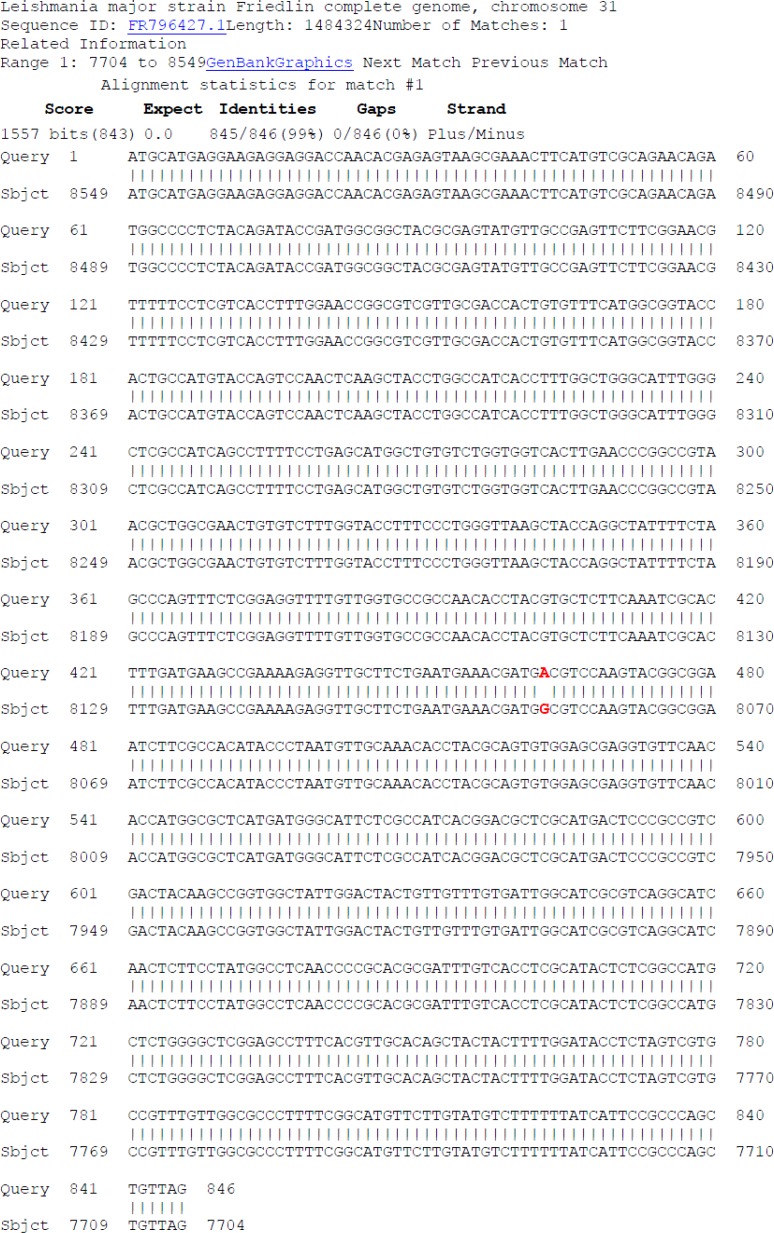
BLAST analysis and comparison alignment of *AQP1* from *Leishmania major* (MRHO/IR/75/ER) with the one in *L. major* Friedlin. The nucleotide change of G to A at position 463 has been shown with red color

The PCR and RT-PCR products showed exactly equal size of *LmAQP1* gene on agarose gel. Molecular characterization of the amplified *LmAQP1* gene at DNA level showed that the coding region of *LmAQP1* (KU514052) contained 846 bp. A mutation was seen at 463 positions resulted in replacement of G with A (Fig. 1).

The homology of *LmAQP1* gene (KU514052) with *LmTRYP6* (AY567835) was 99%. The codon usage analysis showed the followed codons with repeated more than 10 including GCC, TTT, TTC, GGC, CTC, ATG, AAC, TAC. Based on differences between the mentioned gene and those reported previously, it was proposed and accepted in GenBank. The GenBank accession number for the *LmAQP1* sequence is KU514052.

### Primary structure analysis of LmAQP1

The predicted protein encoded by this gene contained 282 amino acids and theoretical pI/Mw of 4.92 / 34000.91. Its homology with LmAQP1 protein from *L. major* Friedlin was 99%. The important feature of this protein is replacement of alanine with threonine at position 155.

### 3D structure analysis of LmAQP1

**Fig. 2:**
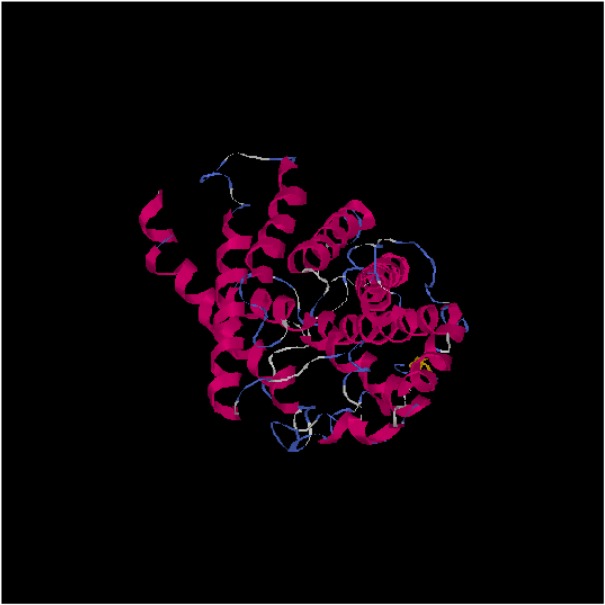
The 3D structure prediction using IT-ASSER database server based on sequence from *L. major* Friedlin strain as a template. The alignment presented amino acid changes with the substitution of alanine with threonine

To predict 3D structure, the mentioned sequence from *L. major* Friedlin strain as a template and the one from Iranian standard strain *L. major* (MRHO/IR/75/ER) sequences were taken to IT-ASSER database server. This server provided alignment, additional with 3D structure. The alignment presented amino acid changes with the substitution of alanine with threonine. This position was not conserved and the mentioned change is common. This change could not affect the 3D structure but may be interfered the protein function. Then, prediction was continued using MOE software for more explanation of the active site and the pore of channel (Fig. 2).

**Fig. 3:**
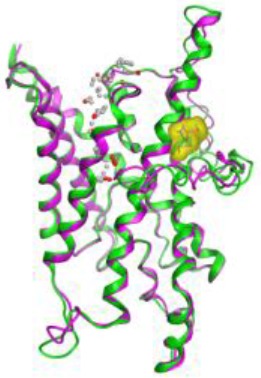
RSMD analysis showed a minimal difference between each atom with 2.5 A. It showed no differences between *LmAQP1* from Iranian strain and the one in European strain (Friedlin)

The amino acid substitution was not present inside the pore. Then, RMSD was calculated and the distance between each atom was measured at 2.5 A° that was very low (Fig. 3). Therefore, no difference was seen between the wild and the mutant strains.

## Discussion

The results of the current study showed that the PCR and RT-PCR products are exactly equal length due to the nature of *Leishmania* genome comprised of interon free. In addition, BLAST analysis showed 99% identity between *LmAQP1* from Iranian (KU514052) and Friedlin (AY567835) *L. major* strains. *LmAQP1* from *L. major* (MRHO/IR/75/ER) showed a point mutation at position 463 with replacement of G with A. Therefore, the encoded protein showed the substitution of threonine with alanine at position 155. Alanine is a nonpolar and an aliphatic amino acid with a simple structure among the amino acids. The methyl group has no role in protein 3D structure, but threonine is a polar amino acid and susceptible to post-translational modification. The hydroxyl chain might undergo O-linked glycosylation as well as phosphorylation through the action of a threonine kinase. Genetic variation in *Leishmania* affects the function of the encoded protein (19) and even introduces a new isolate with different phenotypes (20, 21).

Structural analysis showed a tetrameric complex that each monomer was comprised six transmembrane helices, two minor helices and two conserved (aspargine-alanine-proline) NPA motifs, which present the middle of the pore. The predicted structure is similar to the results of the previous studies (22, 23). One of the important positions of the other AQPs in protozoa is alanine at position 163. This amino acid plays a major role in uptake of water and glycerol, any change in this position alters the water and glycerol transport (24). Interestingly, LmAQP1 has alanine in this position and therefore water and glycerol transportation seem to be normal.

The 3D structure analysis showed that this substitution did not affect the 3D structure. This mutation is one of the common changes that usually happen in nature and does not change the 3D structure. If any other mutation such as replacement of uncommon mutation happened, the viability of the parasite would be affected because as we know this transmembrane protein plays a special role against osmotic pressure. The alteration of A23T in *eotoxin* or *CCL11* does not change the 3D structure of its encoded protein, but it causes the risk of allergic disorders or atherosclerosis (25). In another study, change of A34T inside the light chain immunoglobin gene causes hypercholestromi and systemic amyloidose without any major change in structure (26). This kind of mutation involves in some important and common familial diseases. Therefore, the mentioned point nucleotide change in *LmAQP1* might be a mutation, which generates a different isolate. On the other hand, this kind of mutation that presents in some new generation does not affect the viability of the parasite, but it may change the function of the protein encoded by the gene. This mutation might affect the transport of solutions water and antimonials. This phenomenon has been reported in LmAQP1 from a resistant *L. guyanensis* isolate with G133D in which showed that the 3D structure was normal, but uptake of antimonials changed and therefore the drug concentration inside the parasite was very low (27). Therefore, the current mutation may affect the antimonials uptake and therefore the novel mechanism for drug resistance.

## Conclusion

*LmAQP1* from Iranian *L. major* (MRHO/IR/75/ER) strain has G463A. After bioinformatics analysis, we showed that novel mutation has no effect on 3D structure, but it might affect the protein function and therefore alter uptake of the antimonials. Based on our knowledge, this is the first mutation reported from *LmAQP1*. In this study, we did not investigate challenging of this strain in vivo that would be the authors' recommendations.
